# Oral health reception and risk classification protocol: development and validation in a dental emergency service

**DOI:** 10.3389/frhs.2026.1845431

**Published:** 2026-06-17

**Authors:** Isadora Oliveira de Sousa, Maria de Lara Araújo Rodrigues, Ana Paula Turrioni Hidalgo, Carlos José Soares, Jaqueline Vilela Bulgareli

**Affiliations:** 1 School of Dentistry, Federal University of Uberlândia, MG, Brazil; 2 Area of Pediatric Dentistry, School of Dentistry, Federal University of Uberlândia, MG, Brazil; 3 Area of Operative Dentistry and Dental Materials, School of Dentistry, Federal University of Uberlândia, MG, Brazil; 4 Area of Preventive and Social Dentistry, School of Dentistry, Federal University of Uberlândia, MG, Brazil

**Keywords:** dentistry, emergency care, oral health, risk classification, triage

## Abstract

This study aimed to develop and validate an Oral Health Reception and Risk Classification Protocol for dental emergencies to optimize care and organize service demands. This methodological study was conducted in four stages: literature review to identify existing models, protocol development, content validation by expert judges, and a pilot study to analyze reliability. The protocol, based on the Manchester Triage System and the Oral Health Network Guidelines of Paraná, was structured in nine items: personal data, history of the current condition, pain, pain classification, vital signs, need for medication, medical history, priority, and risk classification. The pilot study included 80 patients treated at the Dental Emergency Service of the School of Dentistry, Federal University of Uberlândia (PSO-HOUFU). A total of 93.75% of patients reported pain, predominantly spontaneous and intense. The most frequent classifications were yellow risk (50%) and green risk (45%). The average time between arrival and treatment was 1 h 33 min, with a dropout rate of 2.5%. Ten on-call professionals participated in the validation using the Content Validity Index. The Kappa coefficient indicated a 97% agreement. The protocol was considered valid in terms of appearance, clarity, and relevance. It can be safely used by health professionals in dental emergency triage.

## Introduction

Organizing the work process and using planning tools are essential steps for effective health care delivery (1). Implementing access to services through reception with risk classification may improve care, as it involves qualified and humanized listening. Risk classification defines the order of treatment according to the complaint, organizing patient flow and ensuring safe and humanized care (2).

The absence of structured triage systems, as well as clearly defined risk criteria and standardized assessments of pain and suffering, substantially undermines the functioning of emergency care services. In such contexts, care delivery is often organized on a first-come, first-served basis, without appropriate prioritization of the most severe cases. This scenario compromises patient flow, delays the timely identification of high-risk conditions, and contributes to service overcrowding. Conversely, the implementation of risk classification systems guides clinical practice according to the severity of each case, enabling the establishment of waiting times aligned with clinical priority. In this regard, triage constitutes an essential tool for the reorganization and overall improvement of care quality in emergency services (3, 4).

Triage begins with the patient's complaint, directing them to a specific flowchart. Questions are asked to identify the complaint until a response is obtained to define clinical priority, level of urgency, corresponding color, and a waiting time frame. Then, priority is no longer defined arbitrarily or by order of arrival, but rather follows established criteria. Assessing clinical parameters and verifying signs of severity are also part of this process, which concludes with data recording and patient referral to treatment or waiting area. Reassessment during the waiting period may be necessary, such as when reaching the time limit or after administering an analgesic (5, 6).

Currently, dental emergency services are often screened intuitively without a specific methodology. This does not adequately characterize the patient's condition for other health professionals and does not serve as a validation parameter (7). That is even more critical for individuals with reduced mobility, flexibility, motor coordination, or perception, such as older adults, pregnant or lactating women, individuals accompanied by infants, people with obesity, and individuals with physical or mental disabilities, who are entitled to priority care in accordance with Law No. 10,048/2000 (8).

In this context, the Brazilian Ministry of Health recommends the Manchester Triage System. However, this methodology, developed for general clinical situations, does not adequately address the specific needs of oral health care (7, 9). Moreover, limited evidence exists regarding reception and risk classification protocols specifically designed for oral health. Such protocols should provide comprehensive patient care, respect the principle of equity of the Brazilian Unified Health System (SUS), and consider patients’ biopsychosocial conditions, their main complaint, and the reason for seeking care.

This study has an innovative character and aims to develop and validate an Oral Health Reception and Risk Classification Protocol within a dental urgent and emergency service, focusing on optimizing treatment and improving SUS demands. This work also aligns with Goal 3 of the Sustainable Development Goals (SDGs) set forth in the 2030 Agenda. Goal 3 seeks to achieve universal health coverage, ensuring equitable access to essential quality health services (10).

## Materials and methods

The study was conducted at the Dental Emergency Service of the School of Dentistry, Federal University of Uberlândia (PSO-HOUFU). This dental hospital provides dental urgent and emergency services to the population of Uberlândia, MG, Brazil, and the surrounding region. The PSO-HOUFU operates 24 h a day, 7 days a week, treating dental urgent and emergency cases (11).

A methodological study was conducted to develop and validate the Oral Health Reception and Risk Classification Protocol. It included four stages: (1) literature review, (2) development of the Oral Health Reception and Risk Classification Protocol, (3) content validation of the protocol, and (4) a pilot study to analyze reliability.

### Stage 1—literature review

A literature review was conducted between August 2024 and August 2025, with the aim of supporting the development of the dental triage protocol. A narrative review with an exploratory approach was adopted, including articles encompassing all types of study designs, as well as institutional documents, theses, dissertations, and protocols, preceded by a scoping stage to identify relevant terms and organize the body of evidence (12). The search was performed in the PubMed, SciELO, and LILACS databases, using the terms “patient triage,” “emergency service,” “oral health,” “risk classification,” and “dentistry.” Considering the exploratory nature, the strategy was conducted in a progressive and iterative manner, without restriction to the exclusive use of controlled descriptors. Institutional documents and secondary references were also used, with emphasis on the Manchester Protocol (13), the Oral Health Care Network Guideline Protocol (14), and Law No. 10,048 (8).

The findings supported decisions regarding the items that should be included in dental triage. The Oral Health Reception and Risk Classification Protocol was based on its relevance documented in the scientific literature and clinical applicability.

### Stage 2—development of the oral health reception and risk classification protocol

**Figure 1:**
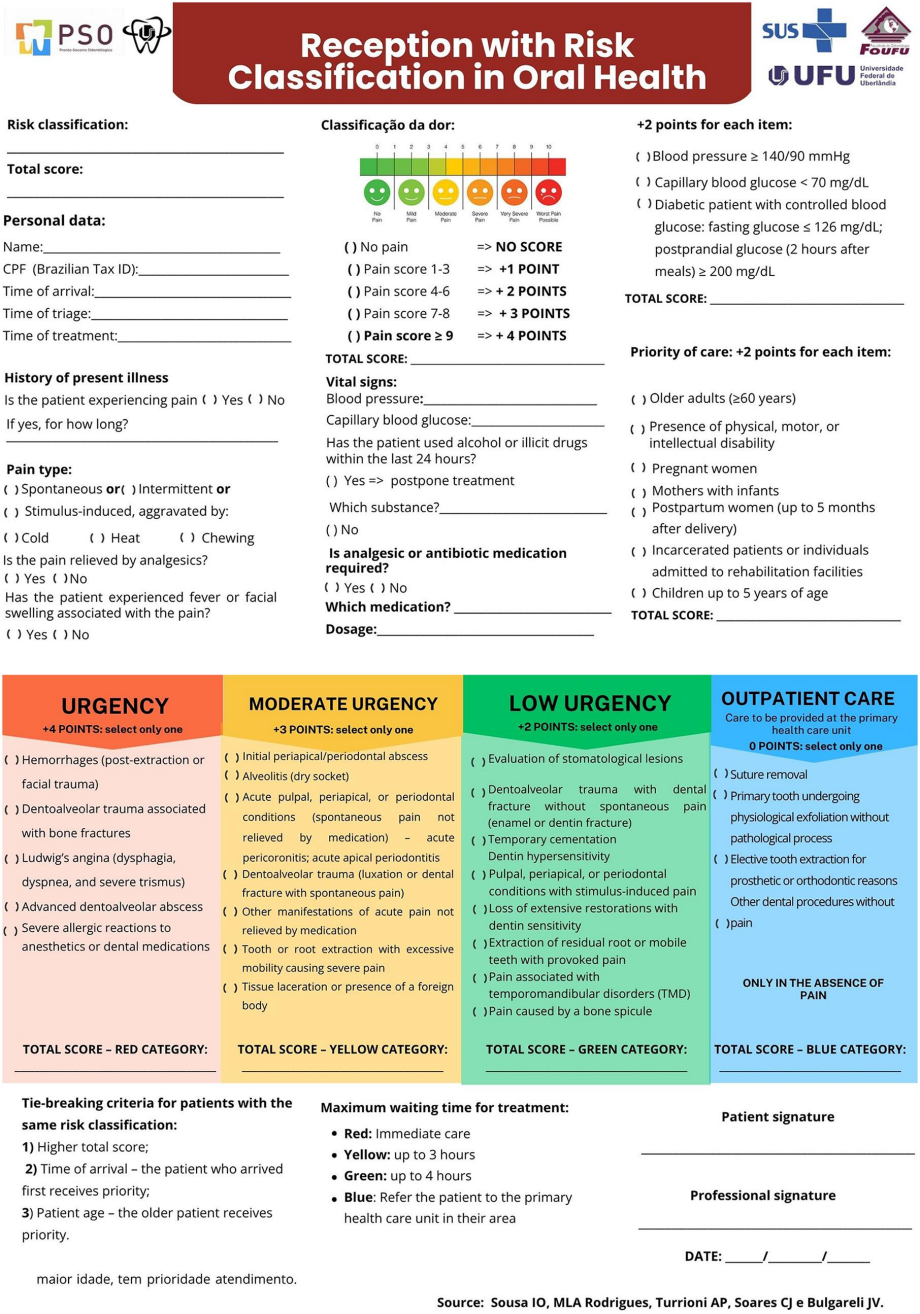
Oral health reception and risk classification protocol.

The protocol was developed based on information regarding the reason for seeking dental care, pain characterization, measurement of vital signs, definition of care priorities as established by current legislation, and patient classification according to risk levels: red (+4 points), yellow (+3 points), green (+2 points), and blue (no score) (Figure 1). It must be completed following the sequential order of items, and each item must be answered to enable the establishment of risk classification at the end.

Its purpose is to classify patients according to their level of risk while simultaneously assigning a score. The final score corresponds to the sum of the scores attributed to each item in the protocol and is considered in conjunction with the risk classification to define care priority. Thus, the protocol allows the determination of priority within the same risk category: when two or more patients fall into the same classification, the scoring system helps identify which patient requires more urgent care. All items in the protocol contribute to this final score.

Tie-breaking criteria were established for situations in which two or more patients present the same risk classification. In such cases, the patient with the highest final score should be given priority. If the final score is also identical, the tie-breaking criterion becomes the time of arrival, with priority given to the patient who arrived first. If a tie persists with the same risk classification, final score, and arrival time, priority is given to the older patient.

The Personal Data item collects the patient's name, SUS Health Card number, as well as the time of arrival at reception, call for triage, and call for treatment. These data are essential for monitoring the patient's actual waiting time. The History of Present Illness item identifies whether the patient is experiencing pain. A positive response indicates the need for urgent or emergency care.

The Pain item aims to classify the pain reported by the patient, assisting in risk classification. When pain subsides with medication, the patient's classification is green. Conversely, if pain persists despite the medication, the classification changes to yellow. If the patient reports fever or facial swelling resulting from pain, the classification is also yellow. The Pain Classification item, in turn, differentiates pain intensity levels among patients. Those presenting more intense pain receive a higher score, consequently increasing their overall score.

The Vital Signs item measures blood pressure and capillary blood glucose to identify possible systemic alterations that may preclude dental care. It also investigates whether the patient has used alcohol or drugs in the previous hours, specifying the type of substance. If the response is positive, treatment is postponed. The Need for Medication item records whether, during reception, the patient required any medication to enable treatment. This medication may include analgesics or antibiotics administered by the team or the recommendation to use the patient's routine medications, such as antihypertensives and hypoglycemic agents, among others.

Questions related to Past Medical History identify any systemic condition that contraindicates treatment continuation. In these cases, blood pressure and blood glucose values should be within the limits allowed for performing dental procedures, according to the measurements recorded in the Vital Signs item. Patients in this condition receive two additional points, increasing their score and, consequently, their priority for treatment. Once the patient meets any of the priority criteria established by Law No. 10,048 (8), the patient receives two additional points for each marked criterion, increasing the final score. This law ensures administrative treatment priority, such as in queues, counters, and public and private institutions. However, in health services, the principle of equity must prevail, guided by reception and risk classification protocols used in the SUS, in which clinical severity overrides the legal order of priority (15).

Finally, Risk Classification organizes the main demands of dental urgent and emergency services. It aimed to develop a categorization that would practically and efficiently reflect the most recurrent situations in urgent and emergency service routines. Each item included in the different classification levels (red, yellow, green, and blue) was carefully analyzed and discussed in meetings with the professional teams of PSO-HOUFU and the research team to ensure its clinical adequacy and practical applicability in the local context. The patient's complaint is marked according to the available options and determines the risk classification. Each risk level corresponds to a specific score: red adds four points; yellow, three points; green, two points; and blue, zero points (Figure 1). Thus, the patient with greater urgency also receives the highest score. Moreover, patients classified as blue risk will not be assisted in emergency services, as their demand does not constitute urgency or emergency. These patients are advised to seek care at a primary health care unit.

### Stage 3—content validation of the protocol

The expert judge committee consisted of dentists, all holding doctoral degrees, selected through convenience sampling of an intentional nature, according to previously defined expertise criteria (16). The participants worked as on-call professionals in dental emergency services, with at least 2 years of experience in urgent and emergency settings, and were affiliated with public services that provide this type of care.

The definition of the number of specialists was based on methodological literature on content validation, which recommends the participation of five to ten judges, considering the characteristics of the instrument, the qualifications of the professionals, and the feasibility of participation. In this context, the composition of the committee with ten specialists was considered appropriate to ensure consistency, representativeness, and rigor in the instrument evaluation process (17, 18).

The data were collected using a structured questionnaire on the Google Forms platform. The Content Validity Index (CVI) was used for quantitative content validation. It measures the proportion of judges who assigned scores of 4 or 5 to the criteria of appearance, clarity, and relevance, based on a 5-point Likert scale. The CVI of each item was obtained by averaging the indices of the three criteria, and the overall CVI of the instrument was calculated by averaging the CVIs of all items. A minimum agreement of 0.80 among judges was considered satisfactory, and items with a CVI below this value were revised (13). Accordingly, the judges evaluated each item using a 5-point Likert scale, considering three criteria: appearance, clarity, and relevance. The scale for the appearance and clarity criteria ranged from 1 (poor) to 5 (excellent), including fair (2), good (3), and very good (4). Regarding relevance, the scale ranged from 1 (irrelevant) to 5 (highly relevant), including slightly relevant (2), moderately relevant (3), and relevant (4) (19, 20).

Items considered inadequate or ambiguous, as well as those that demonstrated better suitability to the proposed context, were modified. New items were also incorporated based on the specialists’ suggestions, while preserving the original structure and objectives of the instrument.

The Kappa coefficient (*k*) was also used to assess inter-rater agreement. This coefficient represents the ratio between the proportion of times the judges agree and the maximum rate of agreement expected by chance (21).

At the end of the validation period, the collected data were analyzed descriptively. The changes suggested by the specialists were progressively implemented until reaching a consensus (22). This process resulted in the final version of the Oral Health Reception and Risk Classification Protocol.

### Stage 4—pilot study for reliability analysis

A pilot study was conducted at the PSO-UFU to analyze the reliability of the Oral Health Reception and Risk Classification Protocol.

Patients underwent a structured triage that included qualified listening to their main complaint, identification of signs of pain and discomfort, and application of a standardized questionnaire containing clinical and sociodemographic variables, such as dental anamnesis, presence of comorbidities, medication use, gestational status, presence of disabilities, reason for seeking care, and reported pain intensity. When indicated, clinical parameters such as blood pressure and capillary blood glucose were measured.

Based on this information, a risk score was assigned using the Manchester Triage System (13) adapted to the dental context, allowing the determination of care priority. The result categorized patients into a color-coded system: red (emergency), yellow (moderate urgency), green (non-urgent), and blue (outpatient cases), which facilitated an immediate visualization of severity and the organization of care demands (14).

Sample size calculation considered an estimated universe of 10,000 visits over 6 months in 2025, using an 80% statistical power and a 5% significance level (*α* = 0.05). The calculation was performed using G*Power software and yielded an estimated sample of 375 patients.

For the pilot study phase, 80 participants were included, corresponding to approximately 21% of the total estimated sample (375). This proportion is consistent with methodological recommendations for pilot studies, which may use reduced fractions of the main sample, often between 10% and 20%, as their purpose is to assess the feasibility, applicability, and initial performance of the instrument, rather than hypothesis testing with statistical power (17, 18).

The study was approved by the Research Ethics Committee of the Federal University of Uberlândia (CAAE: 86258824.5.0000.5152) in accordance with the ethical research guidelines in Human and Social Sciences established by Resolution No. 510 of April 7, 2016, of the Brazilian Health Council.

## Results

The literature review (Stage 1) provided only a few publications on dental triage applied to urgent and emergency services. The studies report local protocols, developed empirically and without scientific validation, which limits their reproducibility and applicability across different care settings.

Nonetheless, these initiatives were essential for guiding the development of the proposed instrument, which aims to standardize and qualify the Oral Health Reception and Risk Classification Protocol.

The Manchester Triage System (13), the Oral Health Care Network Guideline Protocol (14), and Law No. 10,048 (8) (priority of care criteria) were the primary references. They supported the initial structuring of the protocol, comprising clinical urgency criteria, level of complexity, and priority of care.

Regarding instrument validation, the expert committee consisted of ten judges of both sexes (50% men and 50% women), all holding doctoral degrees, and with a mean experience of 9 years in dental emergency services. Three were specialists in restorative dentistry, three in public health/preventive and social dentistry, and one specialist each in endodontics, pediatric dentistry, hospital dentistry, and periodontology.

After an evaluation by the expert committee, all instrument domains reached a CVI ≥ 0.93 (Table 1). This result was achieved after incorporating the specialists’ suggestions, such as changes to the protocol layout, removal of terms that were difficult to understand or that did not contribute to the study, and modification of the sequence of items. Regarding the specialists’ judgment of appearance and relevance, the mean CVI of the items was 1.0; however, the mean item index was 0.80 (Table 2).

**Table 1:** Likert scale results obtained during the consensus phases.

Indicator	Content validity index						
	1. Does the reception and risk classification protocol present an appropriate appearance, with format, organization, and visual language that facilitate its use?		2. Are the information and instructions contained in the reception and risk classification protocol clear and easy to understand for the professionals who use it?		3. Is the content of the reception and risk classification protocol relevant and appropriate to guide the practice of reception and risk classification in oral health?		Judges' area of expertise
Likert scale							
Judges	Clarity – Round 01	Clarity – Round 02	Relevance – Round 01	Relevance – Round 02	Organization – Round 01	Organization – Round 02	Area of expertise
Judge 01	4—Very good	5—Excellent	4—Very good	5—Excellent	5—Highly relevant	5—Highly relevant	Pediatric dentistry
Judge 02	3—Good	5—Excellent	5—Excellent	5—Excellent	5—Highly relevant	4—Relevant	Public health
Judge 03	3—Good	4—Very good	4—Very good	4—Very good	5—Highly relevant	5—Highly relevant	Periodontics
Judge 04	5—Excellent	4—Very good	5—Excellent	4—Very good	5—Highly relevant	4—Relevant	Restorative dentistry
Judge 05	3—Good	5—Excellent	4—Very good	4—Very good	5—Highly relevant	5—Highly relevant	Public health
Judge 06	4—Very good	4—Very good	3—Good	4—Very good	4—Relevant	4—Relevant	Hospital dentistry
Judge 07	3—Good	5—Excellent	3—Good	4—Very good	4—Relevant	5—Highly relevant	Public health
Judge 08	3—Good	5—Excellent	3—Good	3—Good	4—Relevant	5—Highly relevant	Endodontics
Judge 09	5—Excellent	5—Excellent	5—Excellent	5—Excellent	5—Highly relevant	5—Highly relevant	Restorative dentistry
Judge 10	5—Excellent	5—Excellent	5—Excellent	5—Excellent	5—Highly relevant	5—Highly relevant	Restorative dentistry

**Table 2:** Content validity index (CVI)—agreement of the evaluated items.

Evaluation of the items	Evaluated items					
	Appearance		Comprehension		Relevance	
	Appearance – Round 01	Appearance – Round 02	Comprehension – Round 01	Comprehension – Round 02	Relevance – Round 01	Relevance – Round 02
Agreement (items 4–5)	5	10	7	9	10	10
Disagreement (items 1–3)	5	0	3	1	0	0
CVI	0.44	1	0.53	0.8	1	1

All items across the three evaluation aspects achieved a mean CVI ≥ 0.93, with an overall index of 0.97, as assessed by the Kappa coefficient (*k*). The questionnaire provided space for suggestions and improvements for the instrument, which were analyzed descriptively and incorporated into the material (19, 20).

The pilot project was conducted with a sample of 80 patients. The mean time between patient arrival and reception with risk classification was 30 min. The mean time between reception and dental care was 1 h and 3 min. Thus, the total mean waiting time from arrival at the emergency service to dental care was 1 h and 33 min. Only two patients withdrew from treatment while waiting in reception after the triage, corresponding to a 2.5% withdrawal rate.

Regarding the History of Present Illness item, 93.75% (*n* = 75) of patients reported experiencing pain at reception, while 6.25% (*n* = 5) reported no pain. As for the type of pain, 46.25% (*n* = 37) of patients reported spontaneous pain, 37.5% (*n* = 30) reported stimulated pain, 11.25% (*n* = 9) reported intermittent pain, and 5% (*n* = 4) represented missing data.

Furthermore, 45% (*n* = 36) of patients stated that pain did not subside with medication, 41.25% (*n* = 33) reported pain relief with the medication, and 13.75% (*n* = 11) were unable to provide this information. Regarding the Pain Classification item, 5% (*n* = 4) reported the absence of pain, 6.25% (*n* = 5) reported pain intensity between 1 and 3, 17.5% (*n* = 14) between 4 and 6, 25% (*n* = 20) between 7 and 8, and 46.25% (*n* = 37) above 9.

Patients had their blood pressure and capillary blood glucose measured. Only 3.75% (*n* = 3) presented values at the maximum limits allowed for dental care. They received two additional points, increasing their score and, consequently, their priority of care. Regarding the Need for Medication item, no patients required medication during the pilot study.

According to Past Medical History item data, 96.25% (*n* = 77) of patients were healthy or had adequately controlled systemic conditions at the time of reception. Regarding the Priority of Care item, 87.5% (*n* = 70) of patients did not meet the criteria defined by Law No. 10,048, whereas 12.5% (*n* = 10) received priority care. Finally, according to the Risk Classification item, 2.5% (*n* = 2) of patients were classified as red risk, 50% (*n* = 40) as yellow risk, 45% (*n* = 36) as green risk, and 2.5% (*n* = 2) as blue risk.

## Discussion

The absence of a structured Oral Health Reception and Risk Classification Protocol evidences a significant gap in the organization of dental urgent and emergency services. The present study is highly relevant because it proposes an innovative model that significantly transforms care demands, ensuring higher clinical effectiveness and efficiency.

Unlike medicine, where predominant complaints involve procedures such as wound care and medication administration (22), dentistry presents specific characteristics that most often require direct clinical interventions. This specificity results in longer treatment times, reinforcing the need for an appropriate triage protocol to optimize resources, prioritize case severity, and ensure higher quality patient care.

In this context, the Oral Health Reception and Risk Classification Protocol emerges as a triage tool focused on oral health, prioritizing the reason for seeking dental care while also considering aspects such as pain intensity, the presence of comorbidities, and legal priority criteria. In this sense, implementing risk classification, as recommended by the Brazilian Humanization Policy, is a crucial strategy for reorganizing care demands. It allows the definition of waiting times based on clinical severity, promoting humanized care and improving service quality (23).

The principles of the urgent care network, as recommended by the Ministry of Health, stem from the need to ensure uninterrupted care, 24 h a day, at different stages of treatment. These principles aim to provide comprehensive and effective care in various health situations, such as urgencies and emergencies involving acute or acute-on-chronic conditions of clinical, surgical, and traumatological origins, among others (3).

The pilot study performed at PSO-UFU revealed that the mean time for dental care was 1 h and 33 min. This finding indicates that applying the Oral Health Reception and Risk Classification Protocol helped organize patient flow and reduce waiting time. Most patients were classified as yellow risk (50%) and green risk (45%), whose maximum waiting times, according to protocol validation, would be 3 and 4 h, respectively.

It should be noted, however, that comparing these data with other settings is challenging, as the PSO-HC-UFU constitutes a reference service of considerable regional relevance. In the Brazilian context, no other teaching hospitals offering uninterrupted (24-h) dental emergency care, as in the present study, were identified. Although dental care is available in 24-h Emergency Care Units (UPAs), no validated protocols were identified to support this study. In the local context of Uberlândia, following agreements with the municipal administration, these services were discontinued in UPAs, which contributed to an increased concentration of demand at the PSO-UFU (11).

Recent studies have shown that patient and professional satisfaction is essential for service quality and directly related to the application of Advanced Triage Protocols (ATPs). This study contributes to service satisfaction and improvement due to reduced waiting times. Soster (24) reports that satisfaction is compromised in contexts of overcrowding and prolonged waiting periods.

The importance of structuring reception and triage mechanisms with risk classification is evident, as they organize care flow more efficiently. The implementation of a specific oral health protocol at the PSO-UFU responds to the network's guidelines. It increases rationalization of care, prioritizes more severe cases, and contributes to the effectiveness and humanization of urgent dental care, especially in a scenario of high demand and complexity (25, 26).

Studies indicate that pain is the main subjective factor motivating the search for urgent dental care, representing the main complaint of 78.0% of patients (25). Similarly, the present study demonstrates that 93.75% of patients seeking emergency dental care reported pain, with 41.25% stating that analgesics did not relieve discomfort. Toothache compromises essential aspects of an individual's life, such as eating, learning, and leisure. In this sense, using ATPs helps optimize patient management, reduces the length of stay in emergency services, and promotes positive impacts on cost reduction and quality of treatment (24, 27).

The development and validation of the Oral Health Reception and Risk Classification Protocol represent an important contribution to strengthening clinical practice in health care and to the scientific production in the field. The instrument comprises an innovative treatment tool for populations seeking dental urgent and emergency services. Previously, these patients were assisted on a first-come first-served basis, disregarding the principle of equity advocated by the SUS and clinical severity criteria. The developed protocol guides professional conduct during emergency dental appointments, promoting fairer, more organized, and more effective care for the target population (28).

The protocol validation demonstrated significant and representative characteristics of the evaluated construct. The specialists classified it as having very good or excellent appearance, clarity, and relevance. Its development was based on theoretical domains described in studies that assessed the knowledge regarding oral health risk classification. However, unlike previous approaches, the instrument presents rigorously proven validity and reliability (20).

Moreover, the Content Validity Index (CVI) and the Kappa coefficient (*k*) were used to analyze the data, as both are widely employed in the health field. One measures the proportion or percentage of agreement among judges regarding specific aspects of an instrument and its respective items, allowing individual item evaluation and, subsequently, global instrument analysis. The other measures the degree of agreement among evaluators in health research (27, 29).

This study demonstrated that the implementation of the Oral Health Reception and Risk Classification Protocol has the potential to improve the organization of dental emergency and urgent care services by structuring care flow, prioritizing more severe cases, and aligning with the principles of equity of the Unified Health System (SUS), thereby contributing to the standardization of patient reception. However, this study presents some limitations that should be considered when interpreting the results. Among these are the application of the protocol in a single service, which restricts the generalization of findings, and the structural and operational limitations of the PSO-HO-UFU, which may interfere with protocol implementation.

Further studies are required to improve the knowledge on qualitative or quantitative protocol implementation and assess user and professional perceptions after the dental triage. Additionally, studies enabling digital triage using applications or information systems should be conducted.

The present study developed and validated an Oral Health Reception and Risk Classification Protocol motivated by the need to improve the triage process in high-demand services. The protocol proved to be an instrument with evidence of content validity and suitable for guiding dental emergency care, promoting the prioritization of cases according to clinical severity and contributing to a more organized and equitable service.

Implementing this protocol may represent a significant advance in qualifying patient flow in dental emergency services, aligning with the principles of the Brazilian Unified Health System, particularly equity and effectiveness of care.

## Data Availability

The datasets presented in this study can be found in online repositories. The names of the repository/repositories and accession number(s) can be found below: https://repositorio.ufu.br/handle/123456789/47637.

## References

[1] Leal DL Werneck MAF Oliveira ACB. Validation of the oral health version of the instrument for diagnosing the stage of development of the health care network. Rev Pan-Amaz Saude. (2017) 8(4):65–75. 10.5123/S2176-62232017000400011

[2] Ministry of Health, Brazil. Guidelines on Welcoming and Risk Classification in Emergency Services. Brasília: Ministry of Health (2009).

[3] Pereira RA Coelho CFC. Implementation of welcoming with risk classification in the hospital network and its impact on primary health care. Rev Extensão. (2019) 3(1):179–83.

[4] Oredsson S Jonsson H Rognes J Lind L Göransson KE Ehrenberg A. Uma revisão sistemática de intervenções relacionadas à triagem para melhorar o fluxo de pacientes em departamentos de emergência. Scand J Trauma Resusc Emerg Med. (2011) 19:43. 10.1186/1757-7241-19-43 21771339 PMC3152510

[5] Souza CC. Degree of agreement of risk classification of users treated in an emergency department using two different protocols (dissertation). School of Nursing/Federal University of Minas Gerais, Belo Horizonte (2009).

[6] Camilo DGG de Souza RP Frazão TDC da Costa Junior JF. Multi-criteria analysis in the health area: selection of the most appropriate triage system for the emergency care units in natal. BMC Med Inform Decis Mak. (2020) 20:1–16. 10.1186/s12911-020-1054-y 32085757 PMC7035766

[7] Mackway K Marsden J Windle J. Manchester Triage System. 2nd ed. Belo Horizonte: Folium (2018).

[8] Law No. 10,048, of November 8, 2000, Brazil. Establishes priority of care for the persons speified and provides other measures. Official Gazette of the Union, (2000). Available online at: https://www.planalto.gov.br/ccivil_03/leis/l10048.htm (Accessed May 19, 2025).

[9] Ministry of Health, Brazil, Secretariat of Health Care, Department of Primary Health Care. Welcoming Spontaneous Demand. Brasília: Ministry of Health (2013); (Primary Health Care Notebooks, no. 28, vol. 1).

[10] United Nations. Transforming our world: the 2030 agenda for sustainable development. New York: United Nations (2015). Available online at: https://sustainabledevelopment.un.org/content/documents/21252030%20Agenda%20for%20Sustainable%20Development%20web.pdf (Accessed July 11, 2025).

[11] Federal University of Uberlândia. UFU dental emergency service. Uberlândia: School of Dentistry (2024). Available online at: https://www.fo.ufu.br/servicos/pronto-socorro-odontologico-da-ufu (Accessed October 14, 2024).

[12] Heath A Levay P Tuvey D. Literature searching methods or guidance and their application to public health topics: a narrative review. Health Info Libr J. (2022) 39(1):6–21. 10.1111/hir.12414 34850535 PMC9300102

[13] Mackway-Jones K Marshall P Cooper D. Emergency Triage: Manchester Triage Group. London: BMJ Publishing Group (1997).

[14] State Department of Health, Paraná, Superintendence of Health Care. Guideline for Elderly Health. Curitiba: SESA (2018). p. 126. Available online at: https://www.saude.pr.gov.br/sites/default/arquivos_restritos/files/documento/2020-04/linhaguia1.pdf (Accessed May 19, 2025).

[15] Ministry of Health, Brazil, Secretariat of Specialized Health Care. Urgent and Emergency Care Network. Available online at: https://www.gov.br/saude/pt-br/composicao/saes/samu-192/ran (Accessed June 23, 2025).

[16] Stratton SJ. Population research: convenience sampling strategies. Prehosp Disaster Med. (2021) 36(4):373–4. 10.1017/S1049023X21000649 34284835

[17] Lynn MR. Determination and quantification of content validity. Nurs Res. (1986) 35(6):382–5. 10.1097/00006199-198611000-00017 3640358

[18] Hopkins Pepe L Duddy P Golbitz P Kling K Pecoraro N Poindijour M. Content validation for a medical-surgical orientation competency assessment instrument. J Nurses Prof Dev. (2024) 40(5):231–5. 10.1097/NND.0000000000001081 39162371

[19] Nora CRD Zoboli E Vieira MM. Expert validation: importance in the translation and adaptation of instruments. Rev Gaúcha Enferm. (2017) 38(3):e64851. 10.1590/1983-1447.2017.03.64851

[20] Alexandre NMC Coluci MZO. Content validity in the processes of construction and adaptation of measurement instruments. Cien Saude Colet. (2011) 16:3061–8. 10.1590/s1413-81232011000800006 21808894

[21] Soares JEF Soares NLS Freitas BHBM Bortolini J. Validation of an instrument to assess adolescents’ knowledge about leprosy. Acta Paul Enferm. (2018) 31(5):480–8. 10.1590/1982-0194201800068

[22] Oliveira MBMF Fernandes LC Oliveira IE Oliveira RA Rebustini F Mafra ACCN. Development and content validation of a risk classification instrument. Rev Bras Enferm. (2024) 77:e20230502. 10.1590/0034-7167-2023-0502pt 39258610 PMC11382668

[23] De Matos YV Breda D. Profile of patients treated at the Jardim Veneza urgent care unit, cascavel-PR. FAG J Health. (2020) 2(1):56–66. 10.35984/fjh.v2i1.164

[24] Soster C Anschau F Rodrigues NH Silva LGA Klafke A. Advanced triage protocols in emergency services: systematic review and meta-analysis. Rev Lat Am Enfermagem. (2022) 30:e3511. 10.1590/1518-8345.5479.3511 35293563 PMC8966058

[25] Law No. 14,572, of May 24, 2023, Brazil. Amends Law No. 8,080, of September 19, 1990, to regulate the performance of physicians and dental surgeons in home care within the SUS. Official Gazette of the Union, 2023; May 25; 160(100):1. Available online at: https://www.in.gov.br/en/web/dou/-/lei-n-14.572-de-24-de-maio-de-2023-488991098 (Accessed July 10, 2025).

[26] Costa FF Prudente GM Borba ACG Deus S Castilho TC Sampaio RA. Effectiveness of applying the Manchester protocol in risk classification in urgent care units: a systematic review. RSM. (2020) 115(8):668–81.

[27] Alves IM Freitas LTB Lima LR. Profile of dental emergency care provided at university clinics. RECIMA21. (2023) 4(1):e412537. 10.47820/recima21.v4i1.2537

[28] Sanchez TP Borchardt D Tribis L. Equitable care for spontaneous dental demand through characterization and implementation of color-based risk classification, pain scale, and longitudinal clinic management at the Jardim das Palmas Primary Health Care Unit. Rev Bras Med Fam Comun. (2021) 16(43):2756. 10.5712/rbmfc16(43)2756

[29] Souza AC Alexandre NMC Guirardello EB. Psychometric properties in the evaluation of instruments: assessment of reliability and validity. Epidemiol Serv Saude. (2017) 26:649–59. 10.5123/S1679-49742017000300022 28977189

